# Survival chances of a prey swarm: how the cooperative interaction range affects the outcome

**DOI:** 10.1038/s41598-020-64084-3

**Published:** 2020-05-20

**Authors:** Dipanjan Chakraborty, Sanchayan Bhunia, Rumi De

**Affiliations:** Department of Physical Sciences, Indian Institute of Science Education and Research Kolkata, Mohanpur, 741246 West Bengal India

**Keywords:** Biophysics, Computational biology and bioinformatics, Ecology, Physics

## Abstract

A swarm of prey, when attacked by a predator, is known to rely on their cooperative interactions to escape. Understanding such interactions of collectively moving prey and the emerging patterns of their escape trajectories still remain elusive. In this paper, we investigate how the range of cooperative interactions within a prey group affects the survival chances of the group while chased by a predator. As observed in nature, the interaction range of prey may vary due to their vision, age, or even physical structure. Based on a simple theoretical prey-predator model, here, we show that an optimality criterion for survival can be established on the interaction range of prey. Very short-range or long-range interactions are shown to be inefficient for the escape mechanism. Interestingly, for an intermediate range of interaction, the survival probability of the prey group is found to be maximum. Our analysis also shows that the nature of the escape trajectories strongly depends on the range of interactions between prey and corroborates with the naturally observed escape patterns. Moreover, we find that the optimal survival interaction regime varies depending on the prey group size and also on the strength of the predator and the prey interactions.

## Introduction

In nature, cohesive group formation has been observed in diverse species ranging from bacterial colony to flocking of birds, swarming of insects, schooling of fishes, huddling of penguins, to name a few^1–10^. Swarm behaviour arises due to a variety of reasons as individual members gain mutual benefits from one another belonging in a group while searching for food, finding new nests, migrating from one place to another, or overcoming various environmental hurdles in general^1,2,10,11,12^. Another major factor of forming a group is thought to be due to predation avoidance, where survival chances in a group turn out to be better than for solitary individuals^3,13,14^. Moving in a large group often dilutes the encounter and increases the overall alertness since many eyes could keep a careful watch for possible danger or a predator attack. It also confuses the predator by making it difficult to focus on any particular member among a large group of prey^15^. However, cohesive movements could also be unfavourable for prey as the predator can then easily track the group and attack them. For example, fish schools are easily tracked and caught by marine predators^16^. Moreover, prey at the boundaries and the trailing ones are more vulnerable for predation, so each prey competes within for the protected position. Competition may also arise due to limited food resources or due to aggression within the group. Thus, there is often found to be a trade-off between staying together versus individual needs. So, prey groups always look for efficient strategies for the survival from predator attacks^17–20^. There are different escape strategies that have been observed in nature. For example, a school of marine fish would scatter fixing the predator at the center or splitting up into subgroups creating visual confusion^14,21^. Besides, on finding a potential threat, animal aggregation often moves closer to reduce the chance of being caught by the predator^22^. Moreover, there are instances of direct escapes where prey straight away runs in the opposite direction to escape from the predator or runs in a random zigzag motion to confuse the predator. It is further observed that prey often interacts within the group to avoid predation by opting a different kinds of swarming patterns like spinning, circling, splitting up into sub-groups, etc^19,20,23–25^. However, it still remains far from clear how the local interactions among swarming prey lead to complex behavioural patterns or how prey optimize their survival chances or influences the predation rates etc.

There are several experimental and theoretical studies which have contributed immensely to understand the emergent behaviours of swarming in living organisms. Considerable efforts have also been made to explain the collective dynamics of prey-predator systems. Detailed studies on escape trajectories of different species under threats show a certain degree of unpredictability in their escape patterns that confuse the predator in the chase^23,25^. Besides, how the size of the prey group affects predator attacks and the success rate, have also been investigated in the field^26^. Also, it has been observed that cooperativity in predator groups significantly increase hunting success up to a specific threshold size of the group^27–29^. However, understanding local interactions within the prey group in the natural field is quite challenging due to the unpredictable nature of the predator attack. In such scenarios, theoretical models further help us to get insights into the complex dynamics of collectively interacting systems. For example, based on self-propelled particle models, collective predation and escape strategies have been explored to provide insights into the predation rate and the catch time of the group^30^. In another simple model of a prey-predator system, it has been shown that prey swarm could easily escape from a weak predator. However, as the strength of the predator increases, the system passes through a transition from a confused state of the predator to chasing dynamics^31^. There are also other models on swarming behaviour of prey in the presence of predators where different force laws between predators are explored^32^. Predator confusion and its effect on reducing the attack to kill ratio has also been studied^5,33^. Another evolutionary model suggests that predator confusion drives the swarming behaviour of prey, and the attack efficiency decreases rapidly when predators visual filed is restricted^34^. Further, the survival of prey has been studied by assigning different sighting radius to the prey and its predators on a square lattice that suggests the importance of optimal sighting range for effective evasion^35^.

Indeed, in the natural scenario, the range of interactions of prey may be limited due to their sensitivity, vision, age, or even physical structure. However, very little has been known about the range of cooperative interactions among prey under a predator attack. It is observed that the prey groups rely on their local interactions to confuse the predator. Coordinating the movement of individuals in a group is important to ensure an escape. In this paper, we investigate the effect of the range of cooperative interactions in a swarm of prey while chased by a nearby predator. Based on a simple theoretical particle-based prey-predator model that incorporates the essential attractive and repulsive interactions between prey and the predator, we study the escape dynamics and the survival probability of the prey group by varying the interaction range among prey under a predator attack. Our analysis shows that the range of cooperative interaction has a strong influence on the escape trajectories of prey. It also hugely alters the survival outcome of the prey group. Cohesive interactions with the entire group or no interactions among prey appear to be unfavourable for their survival. Interestingly, we find that the survival of the group is maximum within intermediate ranges of interaction radius. We further investigate how the optimal survival regime depends on the size of the prey group, the strength of the predator, also on the strength of attractive and repulsive forces among prey-prey and prey-predator. In addition, we also analyze how the spatial correlations among prey and the collective ordering of the group get affected by the change in  the interacting radius.

## Theoretical Model

**Figure 1:**
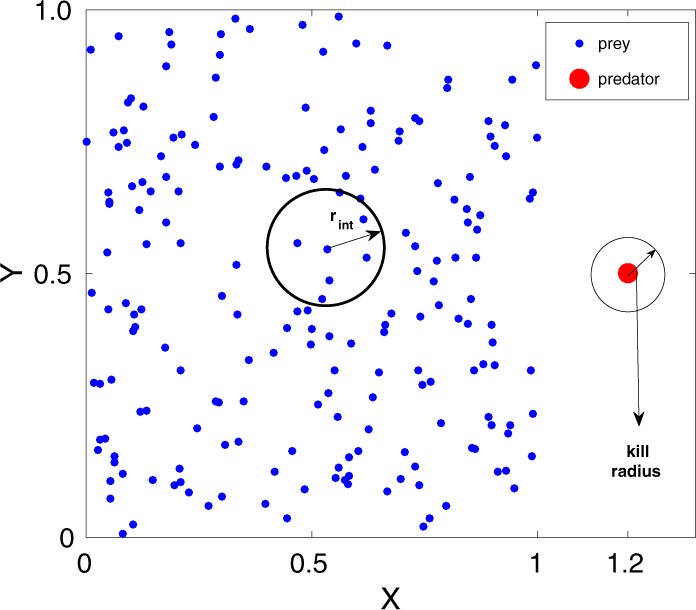
An illustration of the initial configuration of the prey group and the predator. The smaller dots (blue) represent the position of prey and the bigger dot (red) is the position of the predator. Here, ${r}_{{\rm{i}}nt}$ denotes the interaction radius of each prey in the group. The circle shows the kill radius around the predator.

In our model, we consider a group of $N$ prey represented by active particles on a two-dimensional space, as illustrated in Fig. 1. Each prey is characterized by its position, ${\overrightarrow{r}}_{i}$, and velocity, ${\overrightarrow{v}}_{i}$. To mimic the real scenario in the field, we consider that each prey moves in open space following the swarm dynamics. As a swarm is generally organized due to collective interactions among its members, here, we consider that each prey moves based on attractive and repulsive interactions with the neighbouring prey in the group. Moreover, in our study, the prey swarm moves in open space, unlike other studies where periodic boundary condition has been imposed. We focus on the escape dynamics when the prey group is under attack by a nearby predator, as shown in Fig. 1. In general, due to physical or sensory constraints, prey cannot interact with all other prey in a large group at the time of escape^36^. Therefore, we consider that each prey interacts with the neighbouring prey within a certain reaction radius, ${r}_{{\rm{i}}nt}$, for their survival. We model the prey-prey and the prey-predator interactions based on a simple particle-based model where Newtonian type pairwise attractive and repulsive forces have been used^31^. In our theory, each prey interacts with the surrounding prey within the reaction radius by pairwise long-range attraction force and short-range repulsion force. Thus, the prey-prey interaction force for the $i$’th prey is given by averaging over all interacting prey within the reaction radius, ${r}_{{\rm{i}}nt}$,

1 $${\overrightarrow{F}}_{i,{\rm{p}}rey-prey}=\frac{1}{{N}_{{\rm{i}}nt}}\mathop{\sum }\limits_{j\mathrm{=1}}^{{N}_{int}}\left(\beta ({\overrightarrow{r}}_{j}-{\overrightarrow{r}}_{i})-\alpha \frac{{\overrightarrow{r}}_{j}-{\overrightarrow{r}}_{i}}{|{\overrightarrow{r}}_{j}-{\overrightarrow{r}}_{i}{|}^{2}}\right);$$

where ${N}_{int}$ is the number of prey interacting with the $i$’th prey within the given radius, ${r}_{{\rm{i}}nt}$. Here, $\beta$ denotes the strength of the prey-prey attraction, and $\alpha$ is the strength of the prey-prey repulsion. Moreover, as prey always tries to escape from the predator, the prey-predator interaction is modelled as a repulsive radial force,

2 $${\overrightarrow{F}}_{i,{\rm{p}}rey-predator}=-\,\gamma \frac{{\overrightarrow{r}}_{p}-{\overrightarrow{r}}_{i}}{|{\overrightarrow{r}}_{p}-{\overrightarrow{r}}_{i}{|}^{2}};$$

Here, ${\overrightarrow{r}}_{p}$ denotes the position of the predator, and $\gamma$ is the strength of repulsive interaction between the prey and the predator. On the other hand, as the predator chases the prey group, it could track all prey, and its motion is governed by the attractive force averaged over all survived prey given by,

3 $${\overrightarrow{F}}_{{\rm{p}}redator-prey}=\frac{\delta }{N}\mathop{\sum }\limits_{i\mathrm{=1}}^{N}\frac{{\overrightarrow{r}}_{i}-{\overrightarrow{r}}_{p}}{|{\overrightarrow{r}}_{i}-{\overrightarrow{r}}_{p}{|}^{3}};$$

where $\delta$ signifies the strength of the predator. The predator-prey attractive force dominates over the prey-predator repulsive force when the predator comes significantly closer to the prey because of the cubic term in the denominator of ${\overrightarrow{F}}_{{\rm{p}}redator-prey}$ compared to the quadratic term in case of the prey-predator repulsive force, ${\overrightarrow{F}}_{i,{\rm{p}}rey-predator}$. On the other hand, if the predator starts far away from the prey group, the predator-prey attractive force decays as the distance between the prey and the predator increases. Hence, the prey-predator repulsive force becomes stronger than the predator-prey attraction. In this case, thus, the prey could outrun the predator and survive. Therefore, these competing forces come into play only when the predator starts chasing from close proximity to the prey group. Further, we assume that when the prey comes close to the predator within a certain kill radius, as illustrates in Fig. 1, the prey is killed. Here, we note that the prey-predator interaction could also be considered by different power laws, as discussed by Chen and kolkolnikov^31^. Now, the equations of motion of the prey and the predator can be described by,

4 $$\mu \frac{d{\overrightarrow{r}}_{i}}{dt}={\overrightarrow{F}}_{i,{\rm{p}}rey-prey}+{\overrightarrow{F}}_{i,{\rm{p}}rey-predator},$$

5 $${\mu }_{{\rm{p}}d}\frac{d{\overrightarrow{r}}_{p}}{dt}={\overrightarrow{F}}_{{\rm{p}}redator-prey}\mathrm{}.$$

Here, $\mu$ and ${\mu }_{{\rm{p}}d}$ represent the coefficient of the viscous drag experienced by the prey and the predator, respectively. In our model, for simplicity, we consider the dynamics in the overdamped limit.

We study the prey-predator dynamics in dimensionless units. Position variables are scaled as, ${\overrightarrow{R}}_{i}={\overrightarrow{r}}_{i}/{l}_{0}$, ${\overrightarrow{R}}_{p}={\overrightarrow{r}}_{p}/{l}_{0}$ and the interaction radius as, ${R}_{{\rm{i}}nt}={r}_{{\rm{i}}nt}/{l}_{0}$. The dimensionless time is defined by $T=t/\tau$; where ${l}_{0}$ and $\tau$ represent the characteristic length and time scale of the prey system. Also, the other relevant scaled parameters are given as, ${\alpha }_{0}=\alpha /\tau \mu$, ${\beta }_{0}=\beta /\tau \mu$, ${\gamma }_{0}=\gamma /\tau \mu$, and ${\delta }_{0}=\delta /{l}_{0}\tau {\mu }_{{\rm{p}}d}$.

## Results

**Figure 2:**
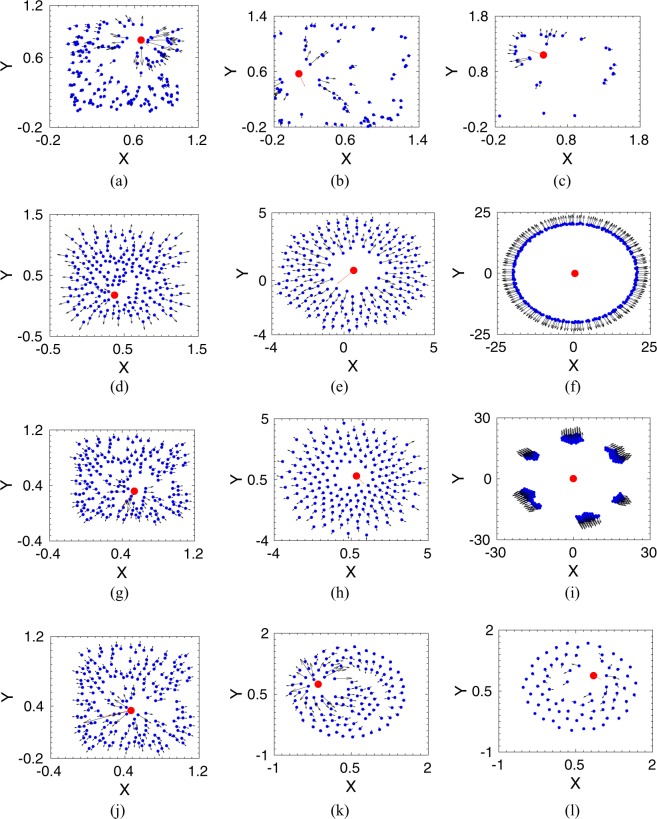
Escape patterns of the prey group (shown by blue dots) under a predator attack (red dot) for different interaction radius, ${R}_{{\rm{i}}nt}$. (**a**–**c**) Snapshots of prey group and the predator at different simulation time, $T=\mathrm{0.1,1,2}$ for ${R}_{{\rm{i}}nt}=0$, i. *e*., there is no interaction among the prey group. (**d**–**f**) Ring formation around the predator at ${R}_{{\rm{i}}nt}=0.5$; snapshots are at $T=\mathrm{0.1,10,1000}$. (**g**–**i**) Splitting up into smaller groups at ${R}_{{\rm{i}}nt}=1.2$; snapshots are at $T=\mathrm{0.1,10,1000}$. (**j**–**l**) For long range interaction, shown here for ${R}_{{\rm{i}}nt}=2.0$, chasing dynamics of the predator at $T=\mathrm{0.1,10,20}$.

We have studied the prey-predator dynamics by solving the coupled Eqs. (4) and (5) numerically. We have investigated the escape dynamics for a wide range of parameter values by varying the prey group size $N$, interaction radius ${R}_{{\rm{i}}nt}$, and also the strength of interactions between prey and the predator. Here, we present the dynamics for a case of a ‘strong’ predator. The ‘strong’ predator signifies that if the prey interacts with all prey to escape from the predator, then the whole group is killed by the predator. On the other hand, in the case of a ‘weak’ predator (when the predator strength, ${\delta }_{0}$ is small or the prey-predator repulsive strength, ${\gamma }_{0}$ is large), the whole prey group could run fast enough and easily escape. The representative parameter values are kept at ${\alpha }_{0}=1.0$, ${\beta }_{0}=1.0$, ${\gamma }_{0}=0.2$, and ${\delta }_{0}=2.5$. In our simulations, we consider that a group of $N$ prey is initially positioned randomly within a square box of unit area, and the predator starts chasing from just outside the box, as illustrated in Fig. 1. The kill radius of the predator is taken as $0.01$. A variety of escape patterns emerge as we vary the range of interaction radius, ${R}_{{\rm{i}}nt}$, among prey as shown in different snapshots in Fig. 2.

We first consider the scenario when prey does not interact with each other, *i.e*., ${R}_{{\rm{i}}nt}=0.0$, and every individual runs away from the predator due to the prey-predator repulsive interaction. As seen  in Fig. 2(a–c), the predator hunts down the randomly moving prey, and the whole group gets caught over time. Now, as we incorporate the prey-prey interactions in the group, we find that for a short-range of interactions radius, the whole prey group is also eventually chased down by the predator. However, as the interaction radius increases, different escape patterns emerge. For example, at the interaction radius, ${R}_{{\rm{i}}nt}=0.5$, as shown in Fig. 2(d–f), interacting prey form a circle surrounding the predator. Thus, the predator gets confused about which direction to attack, and meanwhile, the prey group moves away by circling the predator. Such escape routes of ring formation have also been observed in nature for several cases^21,31^. Other escaping trajectories also arise by varying the interaction radius further, for example, for ${R}_{{\rm{i}}nt}=1.2$, the prey group splits into smaller subgroups and migrates away from the predator in small groups as could be seen from Fig. 2(g,i). The number of subgroups formation depends on the interaction radius, on the size of the prey group, and also on the initial configurations of the group. On the other hand, at an even larger interaction radius at ${R}_{{\rm{i}}nt}=2.0$, as shown in Fig. 2(j–l), chasing dynamics is observed. The predator can catch the prey and eventually chase down the whole prey group as it has been observed when prey interacts with all in the group to avoid the predator.

**Figure 3:**
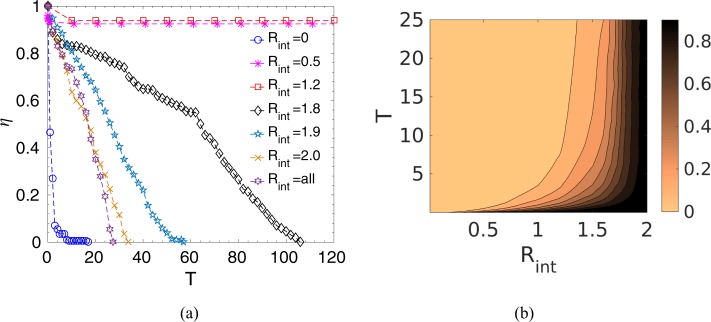
(**a**) Survival probability, $\eta$, of the prey group as a function of time, $T$, for different interaction radius, ${R}_{{\rm{i}}nt}$, and also for the case when all prey interact with each other, ${R}_{{\rm{i}}nt}={\rm{a}}ll$, keeping the predator strength constant at ${\delta }_{0}=2.5$. (This is a representative plot and the same initial configuration has been used to simulate the time evolution for different values of ${R}_{{\rm{i}}nt}$). (**b**) Contour plot of the number of interacting prey on an average within an interaction radius, ${R}_{{\rm{i}}nt}$, normalized by the surviving prey number at that instant as a function of time, *T*, keeping the predator strength, ${\delta }_{0}=2.5$. (The color bar indicates the normalized value of the average interacting prey number *, e.g*., the value ‘1’ means that each prey on an average is interacting with the entire existing group whereas ‘0’ means non-interacting prey).

Now, to quantify the survival of prey as a function of interaction radius, we have calculated the survival probability, $\eta$, of the prey group defined by the ratio of the number of survived prey, ${N}_{{\rm{s}}ur}$, at any instant $T$ to the initial number of prey, *i.e*., $\eta =\frac{{N}_{{\rm{s}}ur}(T)}{N}$. It could be seen from Fig. 3a, as time progresses, for very short-range and long-range interactions, the whole prey group is killed by the predator. However, in the intermediate range, though initially, some prey is caught, but after some time, $\eta$ reaches a steady value, which signifies that most of the prey in the group could escape. This strong dependence on the interaction range could be understood from the competing forces that govern the prey-predator dynamics. With no interaction, *i.e*., *R*_*int*_ = 0 or with smaller interaction radius, as the prey-prey cooperative interaction is not significant, each prey moves somewhat randomly and tries to escape from the predator driven by the prey-predator repulsive force; however as the predator-prey attractive force is stronger, the prey group is chased down and eventually caught by the predator. On the other hand, in case of a very large interaction radius, most prey interact with each other; hence, the whole prey group moves cohesively. As a result, the predator could easily track the whole prey group and hunt them down. Interestingly, in the intermediate range of interaction zone, the initial transient motion shows the chasing dynamics by the predator, however, as time progresses, the local interactions of prey eventually establish coordinated movements to confuse the predator by forming a circle or splitting up into subgroups, or by other escape routes to survive in the long run. In this case, in the beginning, predator-prey attractive force dominates over other forces due to the close proximity of the predator and the prey group. Thus, initially, chasing dynamics have been observed, and the predator is able to catch some prey. However, as time proceeds, local cooperative interaction among prey and prey-predator repulsion helps the prey group to move away, encircling the predator. With the increase in distance between the prey and the predator, prey-predator repulsive force gets strong enough to overcome the attractive force of the predator, and thus, the prey group survive. Such survival strategies of predator confusion have also been observed in nature, for example, the hunting behavior of wolves shows that they eventually give up their pursuit after some initial runs (after failed attempts)^37^. Our analysis, thus, indicates that a certain threshold number of interacting prey is required for coordinated movement to escape away from the predator. Figure 3b shows how each prey on an average interacts with the number of an existing fraction of the population within a given interaction radius while on the chase. As seen, for smaller radius, the interacting prey number is very small; the number increases with increasing ${R}_{{\rm{i}}nt}$, and after a certain threshold radius, each prey interacts almost with the entire group.

**Figure 4:**
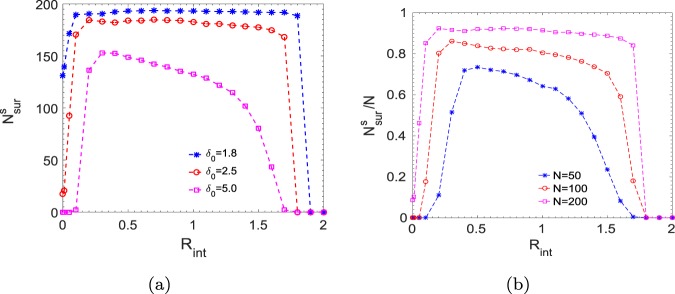
(**a**) Number of survived prey (${N}_{{\rm{s}}ur}^{s}$) as a function of the interaction radius, ${R}_{{\rm{i}}nt}$, for different predator strength, ${\delta }_{0}=1.8$, $2.5$, and $5.0$ keeping initial group size $N=200$. (**b**) Fraction of survived prey number, (${N}_{{\rm{s}}ur}^{s}/N$), as a function of the interaction radius, ${R}_{{\rm{i}}nt}$, for different initial prey group size, $N=\mathrm{50,100}$, and $200$ keeping ${\delta }_{0}=2.5$.

Further, we study the number of survived prey at steady state, ${N}_{{\rm{s}}ur}^{s}$, as a function of interaction radius, as shown in Fig. 4. We have simulated the dynamics for a long time ($T=2000$) to ensure that it has reached a steady-state so that the number of survived prey does not change over time. Moreover, averaging has been done over two hundred such simulation results starting from different initial configurations. It can be seen from the plot that very short-range and long-range interactions are unfavourable for the prey group survival, however, within an intermediate regime, the survival of the group is maximum. In addition, survival depends on the prey group size and also on the strength of the predator. In Fig. 4(a), we keep the initial number of prey at $N=200$, and vary the strength of the predator, ${\delta }_{0}$. It is observed that stronger the predator, lesser the number of survival because the initial catch by the predator is higher for the stronger predator. So the prey interacts in a larger interaction radius to have an adequate number of interacting prey to initiate a coordinated escape and thus, the lower threshold value of ${R}_{{\rm{i}}nt}$ for the survival of the prey shifts to the larger value with increase in the predator strength. An upper threshold value of ${R}_{{\rm{i}}nt}$ is determined by the range where each prey starts to interact with almost all existing prey in the group. We now investigate the dynamics by varying the initial prey group size, $N$, while keeping the strength of the predator constant at ${\delta }_{0}=2.5$. As seen in Fig. 4(b), survival chances of the group increase with an increase in the prey group size when attacked by a predator of the same strength. Larger the prey group size, the predator gets more confused as it becomes difficult to focus on any particular member among a large group of prey. Hence, it effectively reduces the strength of the predator, and thus, the survival chances also go up as has been observed in different field studies^26,38^. Further, in the case of a smaller prey group size, the number of prey on an average interacting within a certain radius is also small. Hence, prey requires to interact comparatively in a larger radius such that the number of interacting prey would be sufficient to establish a coordinated movement to escape away from the predator. So, the lower threshold value of ${R}_{{\rm{i}}nt}$ for the survival shifts to the higher value as we decrease the prey group size. Moreover, the optimal survival regime also depends on the strength of interactions within the prey group. With the increase in long-range attraction strength (denoted by ${\beta }_{0}$ in our model), the prey group becomes more and more cohesive, and thus, becomes more vulnerable as the predator could easily track and catch the whole group. On the other hand, decreasing the attraction strength, ${\beta }_{0}$, helps the prey group to survive up to a larger interaction radius. (For example, keeping the predator strength constant at ${\delta }_{0}=2.5$, if ${\beta }_{0}$ value is increased from 1 to 2; then the prey group gets killed even at a smaller radius, ${R}_{{\rm{i}}nt}=1.5$. Using the same predator strength, if we reduce ${\beta }_{0}$ to 0.5, all members of the prey group get caught at ${R}_{{\rm{i}}nt}=2.8$.) Further, if we reduce the attraction strength, ${\beta }_{0}\mathrm{=0}$, then prey-prey repulsion and prey-predator repulsion make the prey group scatter away and survive. Our study, thus, shows that the survival and the escape dynamics depend on the nature of interactions of the prey group and the predator.

**Figure 5:**
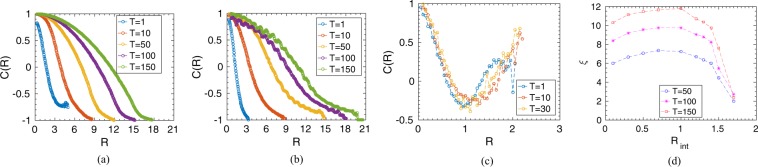
(**a**–**c**) Show spatial correlation in velocity fluctuation, $C(R)$, at different time instances for different interaction radius, ${R}_{{\rm{i}}nt}=0.5$, $1.2$, and $2.0$ respectively. (**d**) Correlation length, $\xi$, as a function of the interaction radius, ${R}_{{\rm{i}}nt}$, for different time. (The predator strength is kept at ${\delta }_{0}=2.5$ and the initial prey group size, $N=200$.).

Moreover, to analyze the collective ordering of the prey group while on escape, we study the spatial correlation in velocity fluctuations as described by^39,40^,

6 $$C(R)=\frac{\sum _{i,j}\delta {\hat{V}}_{i}\mathrm{}.\delta {\hat{V}}_{j}\delta (R-{R}_{ij})}{\sum _{i,j}\delta (R-{R}_{ij})};$$

where $\delta {\hat{V}}_{i}=\frac{\delta {\overrightarrow{V}}_{i}}{|\delta {\overrightarrow{V}}_{i}|}$ is the unit vector along the direction of velocity fluctuation and $\delta {\overrightarrow{V}}_{i}={\overrightarrow{V}}_{i}-{\overrightarrow{V}}_{{\rm{a}}v}$ is the fluctuation in velocity of the $i$’th prey. ${\overrightarrow{V}}_{{\rm{a}}v}$ is defined as the mean velocity, ${\overrightarrow{V}}_{{\rm{a}}v}=\frac{1}{N}{\sum }_{i}{\overrightarrow{V}}_{i}$, and ${R}_{ij}=|{\overrightarrow{R}}_{i}-{\overrightarrow{R}}_{j}|$ denotes the distance between a each pair of prey. Here, $C(R)$ characterizes how the individual prey behaviour deviates from the average behaviour of the group. Figure 5 shows some representative plots of the spatial correlation, $C(R)$, among the prey group for different interaction radius, ${R}_{{\rm{i}}nt}$, at different time instances. As shown in Fig. 5(a,b), within the survival regime of the prey-prey interaction, *e.g*., at ${R}_{{\rm{i}}nt}=0.5$ and $1.2$, the spatial correlation among prey increases with time. However, as shown in Fig. 5(c), for longer interaction range at ${R}_{{\rm{i}}nt}=2.0$, representing the non-survival regime, as all prey interact and move cohesively while chased by the predator, the ordering extends over the entire spatial domain of the group for the whole time period (till the time all prey are killed; here, further time instances are not shown as all prey are killed). We also calculate the correlation length, $\xi$, for which $C(R=\xi )=0$, to measure the average size of the correlated domain within the prey group in the survival regime. Figure 5(d) presents the correlation length, $\xi$, as a function of reaction radius ${R}_{{\rm{i}}nt}$. As seen, $\xi$ increases with increasing ${R}_{{\rm{i}}nt}$ implying prey becomes more and more correlated with increase in interaction radius. However, with further increase in ${R}_{{\rm{i}}nt}$, $\xi$ starts decreasing as the prey group split up into subgroups to escape away from the predator; thus, the correlated domain size decreases.

## Discussion

Here, we show that the range of cooperative interactions in a large group of collectively moving prey is very crucial to strategize their routes of escape under a predator attack. It could be seen from our study, based on a simple theoretical model that accounts for the essential prey-prey and prey-predator interactions, the diverse escape patterns emerge, e.g., ring formation, splitting into subgroups, chasing dynamics, etc. by merely tuning the interaction range between prey. Our study also reveals that the survival chances of the group vastly depend on the local range of interacting prey. As shown, selfish run-away of prey without any interaction is not adequate for the survival; similarly, cohesive movements of the entire group is also unfavourable for the escape. Interestingly, the survival probability is found to be maximum within an intermediate range of interaction radius. Our analysis further elucidates the existence of an optimal interaction regime for survival and a certain threshold number of interacting prey to establish the coordinated movements to escape away from the predator. Within a smaller interaction radius, the interacting prey number is also small, so the local prey-prey interaction force is not sufficient to overcome the strong attractive force of the predator. As the interacting prey number increases with increasing ${R}_{{\rm{i}}nt}$, preys are able to confuse the predator and establish the escape routes. After a certain threshold radius, each prey interacts almost with the entire group so the predator could easily track and chase down the group. The optimal survival regime is further shown to be sensitive to the strength of the predator, the strength of prey-prey interactions, and also on the size of the prey group as it is found in nature. However, under the attack of a weak predator, survival is found to be insensitive to the local interaction range of prey; the whole group could easily escape irrespective of their range of interactions. Further, with purely repulsive interactions, *i.e*., with the prey-prey repulsion and the prey-predator repulsion, prey would scatter away and could escape depending on the relative strength of the predator and the repulsive interactions. However, the prey-prey attractive interaction has a strong influence on the nature of the escape patterns. The collective interactions both due to the attraction and the repulsion among prey eventually determine the emergence of different escape trajectories, whether swarming, chasing, ring formation, or group splitting that has been frequently observed in nature. Moreover, we have also investigated the escape dynamics of the prey group by varying the kill radius of the predator. We find that the effect of varying kill radius is similar to changing the strength of the predator. Further, our study on spatial correlations in velocity fluctuations in prey shows the ordering of the group while on escape. The correlated domain increases with an increase in the interaction range among prey; it reaches a maximum for a certain radius and then again decreases due to splitting up into smaller subgroups. Thus, our simple model could shed light on many aspects of natural prey-predator systems. Such a theoretical framework could further be extended in understanding of other swarm behaviours of various species, for example, during collective foraging, migratory behaviour of birds, or insects, to name a few. This model can also be extended for studying cooperative hunting by multiple predators attacking a prey swarm. Besides, as theoretical modelling and empirical data analysis work hand in hand, more complexity could be incorporated into the model for further quantitative understanding of such conceptual questions in natural scenarios.
